# Determining the Significance of Coronary Plaque Lesions: Physiological Stenosis Severity and Plaque Characteristics

**DOI:** 10.3390/jcm9030665

**Published:** 2020-03-02

**Authors:** John-Ross D. Clarke, Freddy Duarte Lau, Stuart W. Zarich

**Affiliations:** 1 Department of Internal Medicine, Yale-New Haven Health/Bridgeport Hospital, Bridgeport, CT 06610, USA; freddyduartelau@gmail.com; 2 The Heart and Vascular Institute, Yale-New Haven Health/Bridgeport Hospital, Bridgeport, CT 06610, USA; dr.stuart.zarich@bpthosp.org

**Keywords:** fractional flow reserve, coronary artery disease, atherosclerosis, plaque characteristics

## Abstract

The evaluation of coronary lesions has evolved in recent years. Physiologic-guided revascularization (particularly with pressure-derived fractional flow reserve (FFR)) has led to superior outcomes compared to traditional angiographic assessment. A greater importance, therefore, has been placed on the functional significance of an epicardial lesion. Despite the improvements in the limitations of angiography, insights into the relationship between hemodynamic significance and plaque morphology at the lesion level has shown that determining the implications of epicardial lesions is rather complex. Investigators have sought greater understanding by correlating ischemia quantified by FFR with plaque characteristics determined on invasive and non-invasive modalities. We review the background of the use of these diagnostic tools in coronary artery disease and discuss the implications of analyzing physiological stenosis severity and plaque characteristics concurrently.

## 1. Introduction

Angiographic assessment of epicardial coronary lesions has been widely accepted as an incomplete evaluation of the significance of coronary plaque lesions [1]. The traditional anatomic-guided revascularization approaches have been largely superseded by an increasing focus on the functional importance of coronary stenoses [2].

The last two decades of prospective trial data have extensively investigated the impact of revascularization decision-making using fractional flow reserve (FFR) measurements. This physiologic-guided approach to revascularization has shown positive outcomes in many hard clinical endpoints, with benefits being sustained at >5 years of follow-up [3,4,5]. Despite the superior performance of FFR over anatomic strategies in percutaneous coronary interventions (PCI), there are still deficiencies in its prognostic ability, particularly in patients with intermediate lesions and “non-significant lesions”, i.e., FFR values >0.80 [6]. Since the pressure-derived FFR physiologic tool provides information at the lesion level, an advantage over many of its counterparts, its results can be correlated with plaque characteristics obtained on both invasive and non-invasive imaging [7]. The morphologic characteristics of atherosclerotic plaque lesions are independent predictors of adverse cardiovascular events [8,9,10]. The relative merits of physiological stenosis severity measurement over plaque morphology evaluation, and the value of combining both modalities in decision making and risk assessment, are all under study.

In the review that follows we will (i) briefly discuss some of the key concepts underlying the physiologic measures of myocardial ischemia and plaque ‘vulnerability’, (ii) highlight the available evidence for physiologic-guided revascularization strategies and (iii) review the association between physiological stenosis severity by FFR and plaque morphology evaluation and the prognostic implications of using these adjunctive techniques in combination.

## 2. The Significance of Coronary Artery Atherosclerotic Disease

Myocardial ischemia was traditionally solely attributed to anatomically significant narrowing at the level of epicardial vessels [11]. However, the complex interactions of structural and functional abnormalities along the entire coronary vascular tree to myocardial ischemia continue to inform our: (i) definition of ‘coronary artery disease’, (ii) approach to diagnosis, (iii) management strategies and (iv) prognostic measurement [11,12,13]. The more aptly termed ‘ischemic heart disease’ (IHD) describes the clinical symptoms and consequences that result from the combined effects of traditional large-vessel coronary narrowing and the more recently appreciated microcirculatory disease (coronary microvascular disease (CMD)) [12]. The two entities of macrovessel atherosclerosis and CMD are linked pathophysiologically, often co-exist, and manifest in a variety of clinical phenotypes with unique implications [12,14,15,16]. This review will focus on physiologic and structural macrovascular atherosclerotic disease assessment and outcomes.

### 2.1. The Coronary Supply and Myocardial Demand Relationship

**Figure 1:**
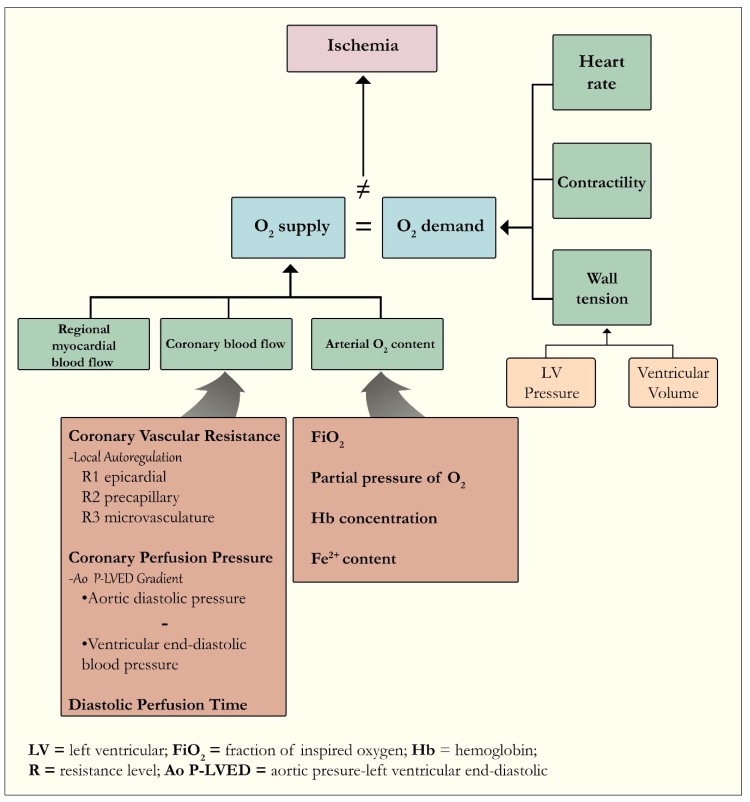
The relationship between myocardial oxygen supply and demand.

Coronary blood flow is closely related to myocardial oxygen requirements (Figure 1) [17,18]. In non-pathologic states, the coronary vascular tree can meet the demands of myocardial muscle even at high workloads, through a variety of regulatory mechanisms, including vasodilation of epicardial vessels [19]. The major regulator vessels of the coronary vasculature are the pre-arterioles and intramural arterioles, which maintain constant blood flow over a wide-range of coronary perfusion pressures through changes in their diameter [20]. When this oxygen supply/demand relationship is mismatched, myocardial tissue metabolic needs are outstripped and the cascade of ischemia begins, with angina being one of the earliest clinical heralds [21,22]. Two metabolites which regulate myocardial blood flow in normal states are carbon dioxide and reactive oxygen species [17]. When myocardial oxygenation is impaired, the low partial pressure of oxygen within the blood itself and/or resultant metabolites, e.g., adenosine, ATP, nitric oxide, prostaglandins, or protons, can impair the inherent regulatory/resistance capability of the vascular supply [17]. When large enough territories supplied by an epicardial vessel are compromised, ongoing ischemia can lead to the clinical entities of contractile dysfunction, hypotension, heart failure, and shock [23].

The many factors which contribute to oxygen supply/demand mismatch can be categorized into coronary and non-coronary mechanisms [24]. When coronary artery disease is present, the extent of atherosclerotic plaque burden (traditionally measured by angiographic vessel narrowing) has been the main measure of ischemia risk. A full review of the non-coronary mechanisms and the drivers of myocardial oxygen requirements are beyond our scope.

### 2.2. Coronary Artery Disease

Transformative insights into the pathophysiology and risk factors for atherosclerotic disease have been gained in recent years [13]. Atherosclerotic plaque formation was initially thought to be attributed mainly to elevated circulating cholesterol particles. The appreciation of the role that inflammation contributes to atherogenesis has had significant implications for our understanding of this disease process and the development of therapeutic strategies [25].

**Figure 2:**
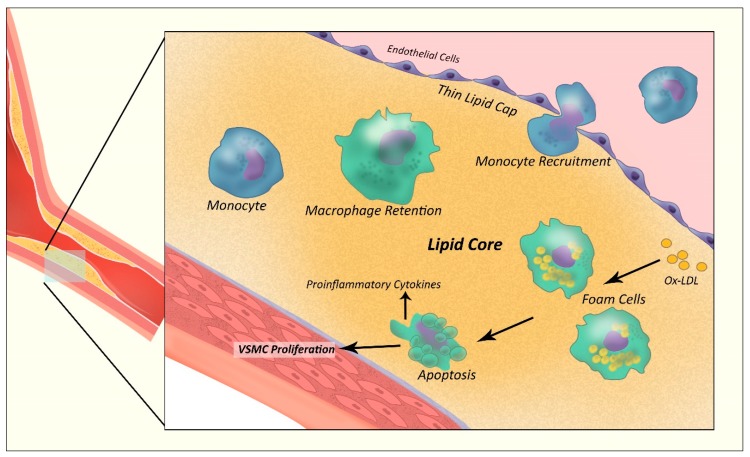
Mechanisms involved in The Generation of a Vulnerable Thin-cap Atherosclerotic plaque. Ox-LDL = oxidized low density-lipoprotein particle; VSMC = vascular smooth muscle cell.

The genesis of the atheromatous lesion requires an initial insult to the endothelial integrity, with an accompanying inflammatory and immunologic response [13,26]. Once vascular endothelial integrity is interrupted, the deposition of lipoprotein within the vessel intima ensues [27]. Macrophage recruitment, matrix metalloprotease release and subsequent lipoprotein mediated apoptosis triggers a cycle of further inflammation, recruitment and accumulation of amorphous material until a necrotic lipid core is formed (Figure 2) [28,29]. These lipid particles undergo oxidation, initiating the release of proinflammatory mediators and directly causing cell necroptosis. This leads to a cycle of cell death, inflammatory cytokine release and immune cell recruitment causing ongoing atherosclerotic plaque propagation [30,31]. The natural history of these plaque lesions include being anywhere along the spectrum of undergoing mineralization, surface angiogenesis and plaque hemorrhage, plaque erosion or rupture [32].

As was alluded to in the introduction of our review, much of the initial view of CAD significance was focused on vessel lumen narrowing being the main predictor of outcomes. The knowledge that a significant burden of atherosclerosis typically exists within the epicardial vessel before it results in compromise of lumen diameter (positive remodeling), has informed our appreciation that other plaque characteristics are better predictors of risk of intracoronary catastrophes [33]. Due to this process of ‘arterial remodeling’, the atherosclerotic lesion propagates outward instead of the intuitive hypothesis of luminal spread [33,34]. As a result of this, significant coronary stenosis is usually a late finding of extensive atherosclerotic burden [35].

#### “Culprit” Plaque versus “Vulnerable” Plaque

Some of the complications which may occur with plaque lesions were mentioned in the previous subsection. These complication events are termed “plaque destabilization” and can manifest clinically in a spectrum from clinically silent events to myocardial infarction or sudden cardiac death.

The most common form of plaque destabilization is plaque rupture [36]. Plaque rupture describes the process of full thickness fissuring of the fibrous cap of the atheroma allowing circulating blood to contact thrombogenic and inflammatory contents from within the necrotic core of the plaque. This release of cytokine mediators commonly results in vessel lumen occlusion and is a major contributor to myocardial infarction and death [32]. Plaque erosion is identified by thrombus formation on the eroded plaque endothelium [37].

The terms “vulnerable” and culprit plaque are prospective and retrospective terms, respectively, used to describe atherosclerotic lesions implicated in plaque complications [26]. The terms have been used inconsistently throughout the literature, but the “vulnerable plaque” was first described in 1988 by Ambrose and Fuster in a retrospective analysis where they described the progression of CAD [38]. The hypothesis was met with optimism as a means of reliably predicting and mitigating clinically significant plaque destabilization events, particularly erosion and rupture. The concept of the vulnerable plaque has many shortcomings in its predictive ability, likely due to the complex milieu of extrinsic factors influencing plaque lesion in vivo [26,32]. Since angiographic stenosis severity has similar shortcomings, a combination of the two factors is likely a better assessor of lesion-specific significance.

### 2.3. High-Risk Plaque Characteristics

Histopathological evaluation of culprit plaques has allowed for various classifications of plaque morphology that likely denote an increased risk of destabilization events [26,32]. These histopathologic features have been correlated with morphology and functional imaging tools (to be described later). In addition, with the advancement of imaging modalities has come the development of high-risk plaque characteristics (HRPC), that could not have been characterized without modern computational tools (see Section 5.1).

High-risk plaques share the features of a thin-cap fibroatheroma (TCFA) [39]. The histologic characteristics of TCFA include but are not limited to: (i) fibrous cap thickness < 65 um; (ii) large necrotic lipid core; (iii) high degrees of inflammation; (iv) positive remodeling; v) neovascularization of the vasa-vasorum and (vi) intraplaque hemorrhage [40]. A few of the histopathological high-risk plaque features are highlighted in Table 1.

### 2.4. Morphological and Functional Assessment Tools

#### Intravascular Ultrasound (IVUS) and Optical Coherence Tomography (OCT)

A comprehensive assessment of plaque vulnerability would ideally include a morphological evaluation of structural characteristics, as well as plaque activity [26]. There are seven clinically relevant intracoronary techniques used in the detection of plaque characteristics. The functional assessment tools include modalities such as: thermography, near infrared (NIR) spectroscopy and radionuclide imaging. This review article will focus on the structural assessment tools: intravascular ultrasound (IVUS) and optical coherence tomography (OCT) and coronary CT angiography (CCTA) (CCTA is covered in detail in Section 5.1). Coronary angioscopy played an early role in identifying the composition of plaque and thrombus, but is no longer routinely employed. [41]

**Figure 3:**
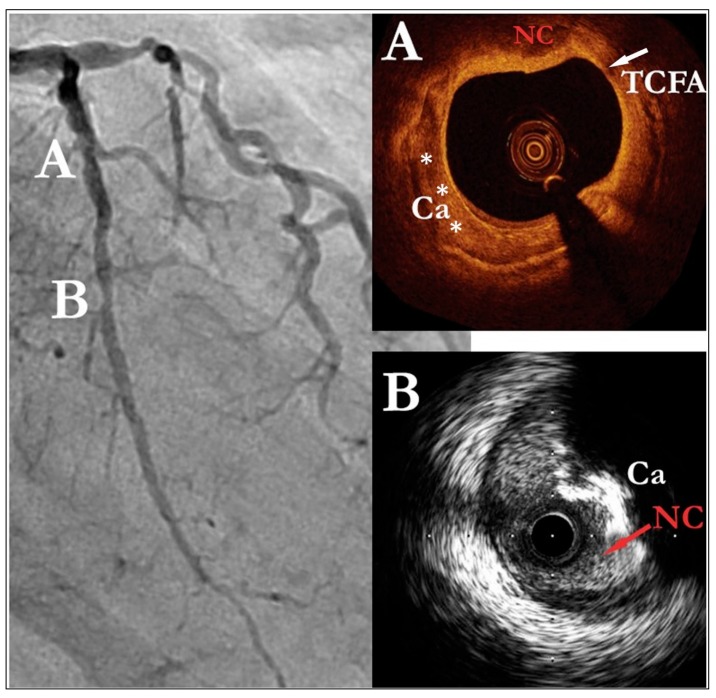
Images of intracoronary imaging modalities. A: Plaque morphology by optical coherence tomography. B: Intravascular ultrasound images. TCFA = thin-cap fibroatheroma; Ca = calcifications; NC = necrotic core.

IVUS was the first invasive plaque imaging technique developed and, along with OCT, remains one of the most widely used modalities (Figure 3) [42]. It consists of a catheter-mounted ultrasound transducer that generates grayscale cross-sectional and linear images with resolutions between 100 and 250 µm. It provides reliable information on lesion density and degree of calcification, allowing for the classification of atherosclerotic plaques into either: (i) soft (plaque echogenicity < adventitia echogenicity), (ii) fibrous (echogenicity between that of soft and calcified plaques), (iii) calcified (plaque echogenicity > adventitia echogenicity), and (iv) mixed (no predominant echogenicity) [43].

IVUS is very effective in detecting positive remodeling and calcification, both major features of TCFA [44]. Due to its inherent poor axial resolution, IVUS can sometimes overestimate fibrous cap thickness [45,46]. The ability of IVUS to detect plaque necrotic cores and neoangiogenesis is promising [47,48].

OCT utilizes near-infrared light in an analogous way to sonography and has the highest resolution of all invasive modalities (5–20 µm) [49]. This allows for better visualization of fibrous caps, collagen content, macrophage presence (a marker of inflammation), neovessels, ruptures and thrombi compared to IVUS. OCT is the only invasive imaging tool that can identify fibrous caps ≤ 65 µm and has shown excellent correlation rates with histopathological analyses [50].

While most aspects of plaque vulnerability are adequately visualized with this technique, the imaging of deeper plaque structures is challenging due to the high scattering of light inherent in this technology, allowing penetration depths of only 1.5–2 mm. This impairs the ability of OCT to properly assess positive remodeling and necrotic core size. Other limitations include the need to displace blood within the vessel with saline flushes (due to its inherent high scattering effect) and a poor discriminatory capacity between areas of calcification and lipid accumulation (both appear as signal-poor areas) [51].

## 3. Principles of the Invasive Physiologic Assessment Tools

### 3.1. Fractional Flow Reserve

Fractional flow reserve is the ratio of the mean distal coronary pressure (*P_d_*) to a stenosis relative to the mean aortic pressure (*P_a_*) measured during maximal hyperemia, whereby [52]

(1) $$FFR=\frac{P_{d}}{P_{a}}.$$

At maximal hyperemia the resistance in the circuit (coronary vessel) is assumed to be constant and minimal, permitting a linear relationship between pressure and flow [53]. FFR represents the fraction of normal coronary blood flow across a stenosis with a normal value of 1 (i.e., *P*_d_ = *P*_a_).

Fractional flow reserve is a reliable and reproducible tool to assess the hemodynamic significance of coronary lesions, and concurrently generates prognostic information [52,54,55,56]. In clinical practice, the sensitivity of FFR has been improved by increasing the threshold value to 0.80 (up from 0.75) [57,58]. This threshold has been almost universally agreed upon by all consensus bodies overseeing PCI guideline recommendations. This, however, creates a “gray-zone” of FFR values in this 0.75–0.80 gap, with unclear clinical significance. The benefit of revascularization in this threshold group has not reached consensus [59,60,61].

### 3.2. Instantaneous Wave-Free Ratio (iFR) and Other Physiologic Tools

The instantaneous wave-free ratio (iFR) provides similar information to pressure-derived FFR [62]. Instantaneous wave-free ratio measures the ratio of *P*_d_ to *P*_a_ during an isolated period of diastole (i.e., the “wave-free period”) [63]. One advantage iFR has over FFR is that it is performed independent of adenosine exposure. The ability to obtain functional assessment of stenoses without the administration of adenosine is valuable in patients with asthma, chronic obstructive lung disease (COPD) and bradycardia, etc., who have contraindications to adenosine use [64]. Unfortunately, the ability of iFR to provide equivalent data to FFR was initially met with reservation due to early non-reassuring data [65,66]. However, numerous investigators have since shown a strong correlation between iFR and FFR measurements in various populations [67,68,69].

When the feasibility of using iFR was assessed in 123 coronary stenoses (78 in non-culprit vessels in a subgroup of patients with acute coronary syndrome and 29 lesions in patients presenting with clinically stable CAD) it was found to correlate significantly with FFR (*r* = 0.78, *p* < 0.001) with a good diagnostic performance (area under the ROC curve = 0.87) [69]. The classification match of iFR was non-inferior in the ACS subgroup compared to the patients with stable CAD [69]. These findings of a high diagnostic performance of iFR were replicated in the first meta-analysis comparing iFR with standard FFR [68]. After analyzing 23 studies including 6381 stenoses, De Rosa et al. noted a correlation of 0.798 (0.78–0.82) between the two measurement tools [68]. Instantaneous wave-free ratio (iFR) was similarly found to have a good diagnostic performance for the identification of FFR-positive stenoses (area under the curve (AUC) = 0.88 [0.86–0.90]; *p* < 0.001). Finally, in a coronary-vessel-specific manner, the correlation and performance of iFR in left main coronary artery (LMCA) stenoses compared to FFR was recently reported [67]. Findings for correlation and performance were *r* = 0.67, *p* < 0.001 and AUC = 0.84; *p* < 0.001 respectively [67].

The two physiologic measures that provide a more dedicated assessment of the microvasculature function are: coronary flow reserve (CFR) and the index of the microcirculatory resistance (IMR) [70,71]. The former modality includes the epicardial vessels. CFR is the ratio of hyperemic to resting absolute flow

(2) $$CFR=\frac{Hyperemic\;Flow}{Resting\;Flow}$$

with a value of >2.0 considered as normal [70]. The index of microcirculatory resistance is a surrogate of the microvascular function, with normal values being >25 [72,73]. Further discussion of the diagnostic performance of CFR and IMR are beyond our scope.

## 4. The Era of Physiological Stenosis Severity Assessment

The role of physiologic-guided PCI in acute coronary syndrome, including STEMI, is an evolving area of research. Ongoing areas of investigation include the use of physiologic guidance in culprit vessels versus complete revascularization either at the index PCI or in a staged procedure. Nonrandomized registry data suggested a potential trend towards increased mortality with the former. The major criticism of the validity of this registry data is its intrinsic susceptibility to selection bias [74]. Analysis of the Harmonizing Outcomes with Revascularization and Stents in Acute Myocardial Infarction (HORIZONS-AMI) trial data suggested a trend towards increased mortality when complete revascularization is performed during the primary PCI procedure rather than in a staged manner [75]. However, more recent metanalyses have shown that complete revascularization at the index PCI may be associated with a decreased risk of major adverse cardiovascular events (MACE), mostly by reducing repeat revascularization [76].

In patients with stable ischemic heart disease, unstable angina and NSTEMI physiologic, rather than traditional anatomic assessment of coronary lesions, has more clearly demonstrated lower short- and long-term mortality and risk of MACE [77,78]. The improved outcomes of multivessel revascularization under FFR-guidance in the Fractional Flow Reserve Versus Angiography for Multivessel Evaluation (FAME) sub-study and Fractional Flow Reserve Versus Angiographically Guided Management to Optimize Outcomes in Unstable Coronary Syndromes (FAMOUS-NSTEMI) trials have supported a Class III to Class IIb change in recommendations for non-culprit revascularization in acute STEMI [79].

### 4.1. Physiologic-guided Culprit Vessel versus Complete Revascularization: Significance and Outcomes

The utility of FFR in the setting of acute STEMI was initially met with apprehension. The hyperemic response in the acute infarct territory is impaired due to acute microvascular dysfunction, leading to a potential false normalization of the FFR measurement. As was previously outlined, the reliability of FFR in epicardial stenosis assumes that there is a negligible contribution from distal microvascular resistance. Notwithstanding this theoretical false-negative risk, many STEMI lesions are hemodynamically significant, with an FFR < 0.80 [80]. In addition, there is a subset of patients with acute STEMI who have evidence of preserved microvascular function (low IMR ≤ 25), that may potentially benefit from invasive physiologic assessment at the time of index procedure [81]. Changes in microvascular function (measured by CFR and IMR) around the time of acute STEMI are a prognostic marker of myocardial recovery and ventricular function [81].

The second question has been whether complete revascularization at the time of primary PCI is an appropriate and safe strategy. Some of the motivation for this is the high prevalence (>50%) of severe stenosis in non-culprit vessels in patients with acute STEMI. These lesions could potentially represent current or future unstable plaque and an increased risk of future ischemic events [82,83]. It would stand to reason that addressing these lesions at the time of primary PCI may lead to improved outcomes. The additional benefits would be a reduction in future catheterization procedures and revascularization need.

The microvascular dysfunction that occurs at the time of acute MI seems to be limited to the infarct-related-artery and is not inclusive of remote myocardial territories, as was previously suspected [84]. FFR can be used to assess non-culprit lesions in the acute setting since IMR is low and does not appear to change much in the post-infarct period [85]. Recent clinical trials have proven the reliability of FFR-guidance in non-culprit vessels in acute STEMI [86,87].

Toma et al. in 2010 found that patients in the APEX-AMI trial who underwent non-infarct-related vessel revascularization had an increased risk of 90-day mortality, heart failure and shock [88] (Table 2). The major limitation is that this trial was conducted in an era when guidelines did not support complete revascularization at an index procedure, and thus only 10% of the cohort underwent non-culprit coronary interventions, possibly leading to a selection bias. Conversely, the Preventive Angioplasty in Acute Myocardial Infarction (PRAMI) trial in 2013 found that performing ‘preventive PCI’ by immediately revascularizing non-infarct vessels after PCI of the infarct artery reduced the combined rate of cardiac death, nonfatal MI and refractory angina by 65% [87]. Of note, in the PRAMI trial an angiographic-guided strategy alone was used in decision making for revascularizing non-culprit vessels.

The COMPARE-ACUTE trial, which was conducted almost half a decade later, investigated the effect of FFR-guided PCI in acute STEMI [86]. Results showed improved MACCE-free survival in patients who underwent complete revascularization rather than culprit-only at the time of index procedure. MACCE, in their report, denoted the composite of mortality from any cause, nonfatal MI, any revascularization, and cerebrovascular events. The secondary seminal outcome from this trial was that intervention was safely deferred in approximately half of the lesions that were anatomically significant, since an FFR was >0.80.

### 4.2. Functional Assessment of Coronary Lesions in Special Populations: Patients with Severe Aortic Stenosis

The prevalence of CAD in patients with severe aortic stenosis is high, and carries unique management implications [91]. Both the coronary plaque and aortic valvular lesion independently have negative effects on the microcirculation [92]. This creates a challenge in identifying the culprit lesion when patients with CAD and severe aortic valvular stenosis present with ischemic symptoms such as chest pain and/or dyspnea [93].

Severe aortic stenosis disproportionately impacts the measurement of hyperemic indices in the catheterization lab [94]. Hyperemic indices have been noted to increase significantly following transcatheter aortic valve implantation (TAVI), whereas resting flow (specifically in the wave-free period of diastole) remains unchanged [94]. The improvement in microcirculatory function following TAVI appears to be independent of the severity of concomitant coronary artery disease [94]. This suggests that, when a patient has both severe AS and CAD, the aortic lesion may be the predominant lesion unless the instantaneous wave-free ratio is ≤0.74.

The evaluation of coronary lesions in patients with severe AS using FFR has been shown to be both feasible and safe [95]. In a study by Pesarini et al. of 54 patients, intracoronary adenosine did not result in any adverse effects during the procedure or at follow-up. Although only minor changes in FFR values before and after TAVI were demonstrated, the findings suggested that functional assessment with FFR may be more reliable if performed after the aortic valve implantation [95].

### 4.3. Coronary Physiology Assessment in the Catheterization Laboratory

The physiology assessment tools used in modern catheterization labs are based on two principles: Doppler velocity and thermodilution. The principles of pressure-derived FFR and iFR were discussed earlier. Considering the guideline recommendations favoring its use in the appropriate settings, FFR assessment is now a part of the interventionalist’s toolbox. The more dedicated microvascular measurement instruments of coronary flow reserve (CFR) and the index of the microcirculatory resistance (IMR) are not as readily available and have not yet made their way into universal practice. Our group has previously discussed a role for more routine use of these more dedicated tools (CFR and IMR) in another review [96].

## 5. The Implications of the Addition of Plaque Characteristics to Traditional FFR Assessment

### 5.1. Noninvasive Plaque Morphology Evaluation: Focus on Coronary CT Angiography

#### 5.1.1. The Evolution of CCTA

Non-invasive modalities like coronary-computed tomography angiography (CCTA) have emerged as alternatives to invasive coronary angiography (ICA) for the assessment of coronary stenosis/luminal narrowing [97]. Since the introduction of 64-slice CCTA in 2005, the computed tomography technology has continued to undergo rapid evolution in its temporal resolution, spatial resolution and volume coverage [98]. CCTA has demonstrated its reliability in diagnosing obstructive CAD in many prospective studies with consistently high sensitivity and negative predictive value (NPV) across various populations [99,100,101].

CCTA has a similar ability to ICA in its detection of measures of luminal narrowing such as: percent diameter stenosis, percent area stenosis, minimal lumen diameter (MLD) and minimal lumen area (MLA) [102]. It can further identify the independent predictors of adverse cardiac outcomes: plaque burden, location and composition [103,104]. Specific high-risk plaque characteristics which can be identified by CCTA are low-attenuating plaques, positive remodeling and plaque calcifications [105]. Additionally, these high-risk features are independent predictors of acute coronary syndrome in patients presenting with acute chest pain and initial negative electrocardiogram and serum troponin [9].

An important clinical question is whether CAD stenosis severity detected by CCTA successfully identifies individuals with myocardial ischemia [98]. Furthermore, does CCTA have the ability to identify an association (if any) between coronary atherosclerotic plaque characteristics and ischemia in a lesion-specific manner [102,106]? The traditional 1- and 2- dimensional CCTA measures of diameter stenosis and area stenosis were found to be inadequate in this regard [102,107]. Nakazato et al. identified a novel 3-dimensional CCTA measure of total arterial plaque disease-percent aggregate plaque volume (%APV), with a better diagnostic ability and discriminatory power in identifying ischemic lesions when compared with other markers of luminal narrowing [102,107]. %APV is calculated by dividing the sum of the plaque area in the vessel wall by the sum of the total vessel volume from the ostium to the distal portion of the lesion [102]. The %APV was greater for ischemic versus nonischemic lesions (48.9% vs. 39.3%, *p* < 0.0001) [102]. The %APV had a superior performance based on receiver operating characteristic curve, with an area under the curve (AUC) of 0.85 compared with diameter stenosis (0.68), area stenosis (0.66), MLD (0.75), and MLA (0.78) [102]. An additional new area of promise is CCTA-derived FFR, which is under current investigation for decision making in stable CAD, as well as acute coronary syndromes (heart flow).

#### 5.1.2. The Link between Plaque Characteristics on CCTA and Hemodynamic Significance by FFR

As was alluded to earlier, the relationship between lesion-specific hemodynamic significance and plaque morphology has been an area of ongoing investigation. Investigators have concurrently used imaging modalities and FFR to gain much-needed insight into lesion characteristics. In the case of CCTA, this has been either with non-invasive CCTA and invasive FFR, or through FFR derived from CCTA (FFR_CT_) [108,109]. A full review of the diagnostic abilities and implications of either FFR methodology is beyond our scope. We will, however, discuss the outcomes when either or both modalities have been used.

The number and type of the various atherosclerotic plaque characteristics (APCs): positive remodeling (PR), low attenuation plaque (LAP) and spotty calcifications (SC) have a direct relationship with lesion-specific ischemia [106]. This has been noted even in lesions that would not meet conventional angiographic classification as ‘severe’. The converse has also been true, whereby the absence of these atherosclerotic plaque characteristics identified lesions with a significantly lower prevalence of ischemia, even in the presence of high-grade stenosis [106].

Two hundred and fifty-two consecutive patients from the Determination of Fractional Flow Reserve by Anatomic Computed Tomographic AngiOgraphy (DeFACTO) study were analyzed via CT imaging, FFR_CT_ and invasive FFR across 407 lesions [110]. The authors noted a 3- to 5-fold increased prevalence of APCs among ischemic coronary lesions. This was similar to the prevalence noted by Park et al. [106]. Additionally, positive remodeling was the only individual APC which provided incremental prediction for lesion ischemia over CT stenosis ≥50% plus FFR_CT_ (AUC 0.87 vs. 0.83, *p* = 0.002) [110].

Rizvi et al., in 2017, retrospectively analyzed the same cohort of patients from the DeFACTO study for the marker- log percent plaque diffuseness (PD) [111] (Table 3). The aim of this was to validate a marker of diffuse CAD beyond the current measures of luminal narrowing and atherosclerotic plaque characteristics (APC). Percent plaque diffuseness (PD) is a per vessel level measure of the sum of all contiguous lesion lengths divided by total vessel length. This underwent logarithmical transformation to obtain log percent PD [111]. Independent of stenosis severity and APCs, every unit increase in log percent PD was associated with a 58% (95% CI: 1.01–2.48, *p* = 0.048) increased likelihood of an abnormal FFR. Area under the receiver operating characteristic curves (AUC) indicated no improvement in discriminatory ability when log percent PD was added to a final parsimonious model of minimal lumen diameter (MLD), PR, and LAP (0.870 vs. 0.867; *p* = 0.33). The authors cautioned that conventional methods like AUC can cause meaningful improvement in reclassification to be overlooked [111], especially when compared with newer statistical metrics like category-free net reclassification improvement (cNRI) which were able to detect improvement by a ratio of 0.21 (95% CI: 0.01–0.41, *p* < 0.001).

#### 5.1.3. Unanswered Questions for Patients with Fractional Flow Reserve > 0.80

We mentioned earlier that, in spite of the superior performance of physiologic tools over anatomic approaches, some patients with non-hemodynamically significant lesions still experience adverse outcomes [5]. This identifies a void which may potentially be filled with adjunctive non-invasive data. Lee et al. in 2019 provided insight into the implications of simultaneous physiological assessment and quantitative and qualitative plaque evaluation in this patient group [105]. Of note, in the FFR ≤ 0.80 group, similar findings to the previously mentioned studies were noted, whereby the number of high-risk plaque characteristics (HRPC) increased with decreases in FFR and both measures independently predicted adverse outcomes at 5 years (expressed as vessel-oriented composite outcome (VOCO) (a composite of vessel-related ischemia-driven revascularization, vessel-related myocardial infarction, or cardiac death)) [105]. The novel finding was that the presence of ≥3 HRPC was independently associated with the risk of VOCO in the FFR >0.80 group.

### 5.2. Intravascular Imaging Techniques: IVUS and OCT

#### 5.2.1. IVUS and FFR

The presence of lipid-rich plaque detected on radiofrequency intravascular ultrasonography (IVUS) correlated with reduced FFR better than plaque area in intermediate lesions [123]. FFR was proportionately lower with increasing size of necrotic lipid cores. Analysis of 350 patients (367 lesions) in the Fractional Flow Reserve and Intravascular Ultrasound Relationship Study (FIRST) study by Waksman et al. sought to determine i) the optimal cutoff for a minimal lumen area (MLA) that correlates with FFR < 0.8 and ii) the usefulness of IVUS MLA as an alternative to FFR to guide intervention in intermediate lesions [116]. FIRST is a multicenter, prospective, international registry of patients with intermediate coronary lesions, defined as 40% to 80% stenosis by angiography. There was a modest correlation between plaque burden and FFR, but no correlation with plaque morphology. This correlation also varied with vessel diameter. Other investigators have noted this modest correlation selectively in main vessel ostial lesions rather than in side branch vessels [115]. When left anterior descending lesions were studied specifically, volumetric quantification of atherosclerotic disease (expressed as intravascular ultrasound (IVUS)-derived percent total atheroma volume [%TAV]) strongly correlated with FFR [117]. Segmental luminal narrowing and total plaque burden were determinants of myocardial ischemia [117].

This inconsistent correlation between IVUS-defined plaque composition or plaque burden and FFR has been noted consistently and has extended to other physiological measures like CFR [118]. The Comparison of Fractional FLow Reserve and Intravascular ultrasound guided Intervention Strategy for Clinical Outcomes in Patients with Intermediate Stenosis (FLAVOUR) trial is a multicenter trial which will hopefully provide clarity on the impact of an IVUS-based strategy compared to FFR-guided PCI on patient outcomes [124].

#### 5.2.2. OCT and FFR

Optical coherence tomography (OCT) outperforms IVUS with regards to reproducibility, and its ability to perform lumen area measurements and delineate the luminal–intima interface [125,126]. The latter two performance metrics are largely a result of higher image resolution with OCT. A few small studies have compared OCT-derived parameters with FFR values [119,120,127,128,129,130]. The OCT-derived parameter which has most consistently correlated with identifying hemodynamically significant lesions has been minimal lumen area (MLA). From these smaller studies, OCT seems slightly superior to IVUS in its ability to detect hemodynamically significant lesions, particularly in vessels <3 mm [119,128]. Usui et al. performed a head-to-head comparison of OCT versus IVUS in their ability to detect functional ischemia across 203 de novo intermediate coronary lesions (186 patients) and noted a similar slight superiority with OCT [121]. The best cut-off values for the prediction of ischemia (FFR < 0.75) were 2.57 mm^2^ (AUC: 0.615, 95% CI: 0.534–0.696), and 1.39 mm^2^ (AUC: 0.732, 95% CI: 0.660–0.804), respectively. In their ability to predict FFR ≤ 0.80, the AUC of OCT-MLA was not significantly greater than that of IVUS-MLA (*p* = 0.13) [121]. Despite the performance of these intravascular imaging modalities, several authors agree that they have not been proven to be reliable substitutes for FFR and that discrepancies between the two modalities (FFR and OCT) should be considered when making OCT-guided revascularization decisions [119,121].

### 5.3. The Future of Coronary Revascularization

The last decade of FFR-guided revascularization trials have provided much insight but have left several questions unanswered [131]. Although many trials have shown reduced risk of revascularizations, they have uniformly failed to reach significance in the endpoint of all-cause mortality. The COMPLETE trial was the first to show a reduction in death from cardiovascular causes and rates of recurrent MI [132]. Two major limitations of this study, however, are the high representation of angiographically significant lesions and the relatively low-risk population [131]. Ongoing trials such as FAME 3 (ClinicalTrials.gov Identifier: NCT02100722), FULL REVASC (ClinicalTrials.gov Identifier: NCT02862119) and RIPCORD 2 (ClinicalTrials.gov Identifier: NCT02892903) will hopefully provide more understanding in this area.

With the advent of CCTA-FFR, we are entering an unprecedented age where lesion significance, plaque burden and plaque morphology can all be assessed concurrently in the non-invasive arena. This will undoubtedly enhance our ability to understand the interaction between “form and function”, i.e., the relationship between plaque characteristics and the degree of ischemia in assessing future coronary events. Invasive techniques will still be required to further our understanding of the role complete versus incomplete revascularization in acute coronary events. We still have much to learn.

## 6. Conclusions

The hemodynamic significance and morphological characteristics of atherosclerotic plaque lesions are both independent predictors of cardiovascular outcomes. Fractional flow reserve allows for the correlation of stenosis severity with both invasive and non-invasive imaging modalities in a lesion-specific manner. The available evidence suggests that the detection of certain high-risk plaque characteristics on imaging correlates with ischemic burden and risk of plaque catastrophes. Although further study is warranted, physiologic and plaque morphology assessment provide additional information and should be used in combination when assessing coronary plaque lesions.

## Figures and Tables

**Table 1:** High-risk features of “Vulnerable” Plaques.

Rupture Propensity Characteristics
Thin cap fibroatheroma morphology
Plaque fissuring
Active inflammation (monocyte/macrophage and sometimes T-cell infiltration)
Presence of superficial calcified nodule
Intraplaque hemorrhage
Endothelial dysfunction
Positive (outward) remodeling
**Erosion Propensity Characteristics**
Endothelial dysfunction
Denudation of endothelial cell layer with underlying thrombogenic milieu ± thrombus formation
Signs of effects of extrinsic factors
≥90% vessel lumen stenosis

**Table 2:** Summary of Landmark Studies using fractional flow reserve (FFR) in Acute Coronary Syndrome/Stable Ischemic Heart Disease.

ST-Segment Elevation Myocardial Infarction (STEMI)-Only Trials						
Study	Year	Study Design	(*n*)	Population	Revascularization Strategy	Outcome
PRAMI	2013	Single-blinded Randomized	465	Patients with acute STEMI and multivessel disease (MVD)	After successful infarct-related artery (IRA) primary percutaneous coronary intervention (P-PCI): randomization to preventive PCI (angiographic assessment of N-IRA) vs. IRA-only	Preventive PCI reduces risk of major adverse cardiac events (MACE)
CvLPRIT	2015	Single-blinded Randomized	296	Patients with acute STEMI and MVD	After successful IRA P-PCI: randomization to complete revascularization (including angiographic assessment of all N-IRAs) or IRA-only	Complete revascularization lowered MACE at 12-months
DANAMI-3—PRIMULTI	2015	Open-label Randomization	627	Patients with acute STEMI and >50% stenosis in ≥1 N-IRA	After successful IRA P-PCI: randomization to FFR-guided complete revascularization vs. IRA-only	Complete revascularization reduced risk of adverse events (mainly through ↓ repeat revascularization)
COMPARE-ACUTE	2017	Randomized Prospective	885	Patients with acute STEMI and >50% stenosis in ≥1 N-IRA	After successful IRA P-PCI: randomization to FFR-guided complete revascularization vs. IRA-only (with FFR assessment of non-culprit lesions)	Complete revascularization reduced risk of MACCE [86]
COMPLETE	2019	Single-blinded Randomized	4041	Patients with acute STEMI and ≥70% stenosis or FFR < 0.80 in ≥1 N-IRA	After successful IRA P-PCI: randomization to staged FFR-guided complete revascularization vs. IRA-only	Complete revascularization was associated with reduced risk of death from cardiovascular causes
**NSTE-ACS and Stable IHD Studies**						
DEFER	2001	Prospective Randomized	325	Patients undergoing elective PTCA for >50% stenosis of native coronary artery	Once FFR > 0.75, patients randomized to deferral vs. performance of PTCA. If FFR < 0.75, then PTCA was performed	No benefit to intervening on non-significant lesions
FAME	2009	Prospective Randomized	1005	Patients undergoing PCI with ≥50% stenosis in ≥2 vessels. (Including non-acute ACS)	Angiographic-guided revascularization vs. FFR-guided PCI (measurement of all indicated stenoses)	Routine FFR-guidance reduces risk of death, non-fatal MI and repeat revascularization
Muller et al. [89]	2011	Single-center Observational Study	730	Patients with SIHD and proximal LAD stenosis 30–70% + other vessel disease <30%	Revascularization vs. OMT based on FFR <0.80.	Medical therapy in patients with non-significant lesions is associated with favorable long-term survival.
Mayo Registry [90]	2013	Single-center Retrospective Registry Study	7358	Patients with NSTE-ACS and SIHD	Performance of FFR-guided PCI in ≥1 vessel vs. Angiography alone.	FFR-guided decision making is associated with freedom from MACE
FAME2	2012	Randomized Placebo Controlled Blinded	888	Patients with stable angina with ≥1 vessel with ≥50% stenosis	Randomized to revascularization vs. OMT. Significant lesions = FFR ≤ 0.80	FFR-guided PCI in combination with OMT is superior to OMT alone in reducing risk of urgent revascularization for ACS symptoms.
RIPCORD	2014	Prospective Cohort Study	200	Patients undergoing elective diagnostic angiogram with ≥1 vessel with ≥30% stenosis.	FFR assessment of all epicardial vessels or major branches of ≥2.25 mm diameter. Significant lesions = < 0.80 (lowest of 2 measurements)	FFR is better at identifying stenoses and influences medical management
FAMOUS-NSTEMI	2014	Single-blinded, Prospective Randomized Controlled Parallel group	350	Patients with recent NSTEMI, ≥1 CAD risk factor, planned PCI within 72h. Diagnostic angiogram with ≥1 vessel with ≥30% stenosis	FFR measurement in all lesions ≥30% stenosis. Revascularization by PCI or CABG vs. medical therapy. Significant lesions = FFR < 0.80	No significant difference in outcomes or quality of life
PRIME-FFR	2017	Nationwide Prospective Study (POST-IT + R3F registries)	1983	Patients having undergone routine use of FFR at the time of diagnostic angiography	Operator discretion of FFR evaluation following angiographic assessment	No difference in MACE in patients with ACS at 1 year. FFR-based deferral was safe
IRIS-FFR [61]	2017	Prospective registry	5846	Patients who underwent FFR measurement of ≥1 coronary lesion.	Revascularization was generally recommended for FFR < 0.75 and deferred with FFR > 0.80. FFR values 0.75–0.80, operator dependent	Revascularization of significant lesions (FFR ≤ 0.75) associated with ↓MACE. OMT for FFR > 0.75 is reasonable.

STEMI = ST-segment elevation myocardial infarction; (*n*) = number of patients; MVD = multivessel disease; MACE = major adverse cardiac events; IRA = infarct related artery; N-IRA = non-infarct related artery; P-PCI = primary percutaneous coronary intervention; NSTE-ACS = non-ST segment-acute coronary syndromes; SIHD = stable ischemic heart disease, FFR = fractional flow reserve, PTCA = percutaneous transluminal coronary angioplasty; OMT = optimal medical therapy; LAD = left anterior descending artery; CAD = coronary artery disease; CABG = coronary artery bypass grafting.

**Table 3:** Studies linking Plaque Characteristics to FFR.

Study	Year	(*n*)	Number of Lesions	Plaque Characteristics	Outcome
CTA					
Nakazato et al. [102]	2013	58	58	Diameter stenosis, MLD, MLA, %APV	%APV enhances identification of ischemic lesions of intermediate stenosis severity compared to diameter stenosis, MLD, and MLA.
Park et al. [106]	2015	252	407	%APV, PR, LAP, SC.	PR is an independent predictor of ischemia for all lesions, while %APV and LAP are only useful in lesions with >50% stenosis.
Gaur et al. [112]	2016	254	484	PV, NCP, CP, LD-NCP, PR	LD-NCP is an independent predictor of ischemia. FFR values were inversely related to PV, irrespective of severity of stenosis.
Nakazato et al. [110]	2016	252	407	PR, LAP, SC	Strong correlation between PR and ischemic lesions. SC or LAP had no significant correlation. PR superior to CT stenosis plus FFR_CT_ for detection of ischemic lesions.
Rizvi et al. [111]	2017	252	407	PD, MLD, PR, LAP	PD may enhance the accuracy of CCTA in detecting ischemic lesions.
Baskaran et al. [113]	2017	249	399	DCV, LPV, NCV, AS	DCV is not an independent predictor of ischemia although it serves as a marker for aggregate LPV, which, in turn, can predict ischemia.
Driessen et al. [114]	2018	208	415	Plaque length, PV, PB, NCP, CP, partial calcified plaque, LAP, PR, SC	NCP, LAP, PR, and SC were independently associated with ischemia.
**IVUS**					
Koh et al. [115]	2012	77	93	MLA, PB, %AS, RI, NR.	Significant correlation between ischemic lesions and MLA, PB, and percent area stenosis.
Waksman et al. [116]	2013	350	367	Total lumen area, proximal lumen area, distal lumen area, %AS	Significant correlation between MLA and FFR. Plaque morphology characteristics have no correlation with FFR.
Jin et al. [117]	2015	130	130	MLA, PB, lesion length, %TAV.	Independent predictors of ischemia included MLA and %TAV.
Brown et al. [118]	2017	89	92	PV, PB, MLA, % fibrous, % fibrofatty, % necrotic core, % dense calcium.	Independent predictors of ischemia included MLA and PB. No significant association between plaque composition and FFR.
**OCT**					
Gonzalo et al. [119]	2012	56	61	MLA, MLD, lesion length, reference lumen area, eccentricity lumen index, % AS.	OCT has moderate diagnostic ability in identifying severe coronary stenoses. It has superior ability compared to IVUS in vessels <3 mm.
Burzotta et al. [120]	2018	40	40	MLA, mRLA, %AS, major plaque ulceration, intracoronary thrombi.	Independent predictors of FFR include MLA, %AS, plaque ulceration, and intracoronary thrombus presence.
Usui et al. [121]	2018	186	203	MLA, %AS.	OCT-MLA is superior to IVUS-MLA in predicting FFR <0.75. However, intravascular imaging is not a substitute for FFR.
Leone et al. [122]	2019	350	446	MLA, pRLA, dRLA, and mRLA.	Early data at 1 month show that patients in the FFR branch underwent less revascularization compared to patients in the OCT branch. Thus, OCT is associated with increased total costs and contrast-induced AKI. No difference in MACE or angina episodes.

(*n*) = Number of patients; MLD = minimal lumen diameter; MLA = minimal lumen area; %APV = aggregate plaque volume percent; PR = positive remodeling; NR = negative remodeling; RI = remodeling index; LAP = low attenuation plaque; SC = spotty calcification; PV = plaque volume; NCP = non-calcified plaque; CP = calcified plaque; LD-NCP = low-density non-calcified plaque; PD = percent plaque diffuseness; DCV = dense calcium volume; LPV = lesion plaque volume; NCV = non-calcified plaque volume; AS = area stenosis; PB = plaque burden; %TAV= % total atheroma volume; mRLA = mean reference lumen area; pRLA = proximal reference lumen area; dRLA = distal reference lumen area.
